# Nucleotide Resolution Comparison of Transcription of Human Cytomegalovirus and Host Genomes Reveals Universal Use of RNA Polymerase II Elongation Control Driven by Dissimilar Core Promoter Elements

**DOI:** 10.1128/mBio.02047-18

**Published:** 2019-02-12

**Authors:** Mrutyunjaya Parida, Kyle A. Nilson, Ming Li, Christopher B. Ball, Harrison A. Fuchs, Christine K. Lawson, Donal S. Luse, Jeffery L. Meier, David H. Price

**Affiliations:** a Department of Biochemistry, The University of Iowa, Iowa City, Iowa, USA; b Department of Internal Medicine and Epidemiology, The University of Iowa, Iowa City, Iowa, USA; c Department of Epidemiology, The University of Iowa, Iowa City, Iowa, USA; d Veterans Affairs Health Care System, Iowa City, Iowa, USA; e Department of Cellular and Molecular Medicine, Lerner Research Institute, Cleveland Clinic, Cleveland, Ohio, USA; Princeton University

**Keywords:** core promoter elements, P-TEFb, PRO-Seq, RNA polymerase II, RNA4.9, cytomegalovirus

## Abstract

The large genome of human cytomegalovirus (HCMV) is transcribed by RNA polymerase II (Pol II). However, it is not known how closely this betaherpesvirus follows host transcriptional paradigms. We applied PRO-Seq and PRO-Cap methods to profile and quantify transcription initiation and productive elongation across the host and virus genomes in late infection. A major similarity between host transcription and viral transcription is that treatment of cells with the P-TEFb inhibitor flavopiridol preempts virtually all productive elongation, which otherwise covers most of the HCMV genome. The deep, nucleotide resolution identification of transcription start sites (TSSs) enabled an extensive analysis of core promoter elements. An important difference between host and viral transcription is that initiation is much more pervasive on the HCMV genome. The sequence preferences in the initiator region around the TSS and the utilization of upstream T/A-rich elements are different. Upstream TATA positions the TSS and boosts initiation in both the host and the virus, but upstream TATT has a significant stimulatory impact only on the viral template. The major immediate early (MIE) promoter remained active during late infection and was accompanied by transcription of both strands of the MIE enhancer from promoters within the enhancer. Surprisingly, we found that the long noncoding RNA4.9 is intimately associated with the viral origin of replication (oriLyt) and was transcribed to a higher level than any other viral or host promoter. Finally, our results significantly contribute to the idea that late in infection, transcription takes place on viral genomes that are not highly chromatinized.

## Abstract (precis)

Human cytomegalovirus infects more than half of humans, persists silently in virtually all tissues, and produces life-threatening disease in immunocompromised individuals. HCMV is also the most common infectious cause of birth defects and the leading nongenetic cause of sensorineural hearing loss in the United States. Because there is no vaccine and current drugs have problems with potency, toxicity, and antiviral drug resistance, alternative treatment strategies that target different points of viral control are needed. Our current study contributes to this goal by applying newly developed methods to examine transcription of the HCMV and host genomes at nucleotide resolution in an attempt to find targetable differences between the two. After a thorough analysis of productive elongation and of core promoter element usage, we found that some mechanisms of regulating transcription are shared between the host and HCMV but that others are distinctly different. This suggests that HCMV transcription may be a legitimate target for future antiviral therapies and this might translate to other herpesviruses.

## INTRODUCTION

Controlling human gene expression at the transcriptional level is accomplished by regulating initiation, elongation, and termination by polymerase II (Pol II). Initiation occurs through the coordinated actions of factors that maintain chromatin around the promoters in an open state and by the general transcription initiation factors. Transcription start sites (TSSs) are thought to be chosen based, in part, on the affinity of components of the initiation factor TFIID for sequences in the −30 region, of the initiator (Inr), and of downstream elements (1). A carefully orchestrated exchange of factors that leads to transient pausing by Pol II at a heterogeneous array of sites producing 5′-end-capped RNAs that average around 45 nt in length occurs immediately after initiation (2–4). Promoter-proximal pausing requires DRB sensitivity-inducing factor (DSIF) and negative elongation factor (NELF), occurs on essentially all expressed genes, and is a necessary intermediate on the path to productive elongation (5–7). Paused Pol II can either terminate or enter into productive elongation (4, 8). The fraction of polymerases transitioning into productive elongation is regulated by P-TEFb, which phosphorylates DSIF (9, 10). This causes a second exchange of factors in which NELF is lost and other productive elongation factors gain control of the engaged polymerase (2, 9, 11). This step can be blocked by treatment of cells with the P-TEFb inhibitor flavopiridol (12). In addition to regulating entry into elongation, promoter-proximal pausing plays a major role in maintaining promoters in an open chromatin state (5, 6, 13) and likely acts similarly at enhancers (14, 15).

Human cytomegalovirus (HCMV) is an enveloped double-stranded DNA (dsDNA) virus with a large linear genome coding for hundreds of proteins (16, 17). The virus replicates in many different human cell types, including primary human fibroblasts, and quickly halts the cell cycle as viral replication begins (18). It persists latently in a nonreplicating state in select cell types until viral replication is induced by specific cellular conditions (19, 20). Upon entering the cell, the capsid of the viral particle is released to dock at the nuclear pore and inject its cargo of naked viral dsDNA into the nucleus. Transcription from the viral major immediate early (MIE) promoter then proceeds to generate mRNAs for viral proteins that are required to activate the expression of viral early RNAs as early as 4 h postinfection (hpi) (21). The early gene proteins include the machinery necessary for viral DNA replication and the expression of late transcription factors (LTFs). Viral DNA replication initiates at a single origin, oriLyt, starting around 24 hpi, and produces long DNA concatemers (22). The combination of viral DNA synthesis and viral LTFs brings about viral late RNA expression (23), which yields proteins that compose the viral particle and modulate the infection. A newly assembled empty viral capsid is stuffed with an individual naked viral DNA genome clipped from the concatemeric DNA intermediate. After the viral particle undergoes further assembly and maturation, it is released from the cell at about 72 hpi.

Because HCMV expresses a number of viral proteins that modulate viral gene expression and has many promoters that have TATT in the −30 region recognized by the LTFs (24), we set out to determine the degree to which the virus coopts the host’s standard transcriptional mechanisms. Several studies have examined the viral transcriptome at various times during lytic infection by HCMV by measuring stable mRNAs (17, 25–27), but a direct examination of transcription is lacking. To fill this gap, we used two precision run-on (PRO) methods, PRO-Seq (28) and PRO-Cap (29), which provide nucleotide resolution identification of the 3′ ends of nascent transcripts in nuclei isolated from cells. Both methods start by extending nascent transcripts in nuclei by one nucleotide in the presence of biotinylated nucleoside triphosphates (NTPs). The biotinylated nascent transcripts are affinity purified using streptavidin beads before ligation to a 3′ adaptor. After a second affinity purification and 5′ adaptor ligation, the RNA is affinity purified a third time. This iterative purification leads to an extremely low background of irrelevant transcripts. PRO-Seq captures all 3′ ends and reveals sites of pausing and the extent of productive elongation across the host and HCMV genome. PRO-Cap requires that the 5′ end of the nascent transcript be capped, and because both ends of the nascent transcripts were sequenced, the data can be used to quantify TSS utilization. Accuracy was enhanced by incorporating an optimized nucleus isolation method that rapidly stops Pol II elongation so that the 3′ ends of nascent transcripts faithfully represent where the polymerase was positioned on the DNA (30).

## RESULTS

### Pol II elongation control is utilized broadly during transcription of the HCMV genome.

**FIG 1:**
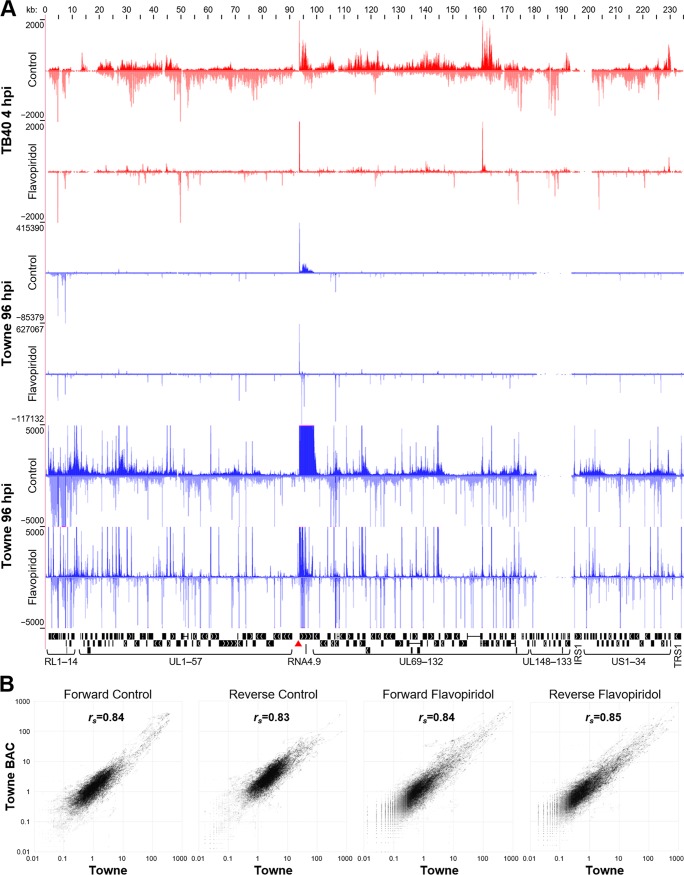
PRO-Seq from HFF infected with HCMV. (A) Nuclei were harvested at 4 h postinfection (hpi) with the TB40E strain (red) or at 96 hpi with the Towne strain (blue). During the last hour, cells were also treated with 0.1% DMSO ± 1 µM flavopiridol. PRO-Seq reads were aligned to the HCMV Towne strain genome (GenBank accession number FJ616285.1) and visualized in a UCSC Genome Browser track hub (top, forward strand; bottom, reverse strand). All tracks range from 0 to the indicated number of reads. Coding regions and the origin of lytic replication (red triangle) are shown below. (B) Pairwise correlation analysis of Towne or Towne BAC PRO-Seq data sets obtained 96 hpi of HFF. Numbers of forward and reverse reads from control and flavopiridol-treated cells were compared for each nucleotide across 212,000 bp of shared genome. Spearman coefficients are indicated.

To determine if early transcription of HCMV genes follows the same transcriptional elongation control paradigm that host genes utilize, PRO-Seq was performed on primary human foreskin fibroblasts (HFF) 4 hpi with a TB40 strain. A pileup of forward and reverse aligned reads almost completely covered the entire HCMV genome in control cells (Fig. 1A). In striking contrast, the results from cells treated with the P-TEFb inhibitor flavopiridol during the last hour of infection to block productive elongation gave only sharp peaks of Pol II transcripts with heights that spanned several orders of magnitude. We have created a track hub for the HCMV Towne assembly, made it available on GitHub (https://github.com/P-TEFb/trackHub_HCMV), and preloaded tracks for all our data sets, related annotations and analyses, and some of the transcriptome sequencing (RNA-Seq) data sets from other labs (see Materials and Methods). With a few clicks, the reader can load this hub in the UCSC Genome Browser, which will allow freedom to navigate the HCMV genome and evaluate our PRO-Seq and PRO-Cap data and all analytical and annotation tracks described below.

To determine if this finding was HCMV strain specific or dependent on the time of infection, PRO-Seq was performed on HFF at 96 hpi with the Towne strain by a similar protocol except that a modification that dramatically improved the potential depth of the reads was introduced. 5′ and 3′ adaptors ligated to the isolated nascent transcripts early in library production each contained 4 random nucleotides. These unique molecular identifiers enable accurate quantification of up to 65,000 molecules of RNA with otherwise-identical sequences. This is especially important for transcripts from paused Pol II, because the transcripts are short and have a limited number of unique 5′ and 3′ ends. If the Genome Browser view is left to auto scale, the broad view of the nascent transcripts over those of Towne at 96 hpi is dominated by several sharp peaks (Fig. 1A). However, if the view is scaled to a maximum height of 5,000 reads, rampant transcription of the genome is again evident. As with the early time point with TB40, flavopiridol focused the transcripts to thousands of discrete locations.

We were surprised to see how pervasive Pol II transcription of the HCMV genome was at 96 hpi. Compared to the 4-hpi data sets, in which only 2% of the total mapped reads were over the viral genome, the 96-hpi data sets exhibited an increase in total mapped reads over the viral genome to 36%. In fact, assuming that there are ∼200 HCMV genomes per cell present at 96 hpi, there is a >100-fold-greater number of reads per base pair for HCMV than for the host. This is partly due to a few extremely highly utilized viral promoters but mainly reflects a greater density of promoters in HCMV. Our initial analysis of deep, high-resolution data supports the idea that most promoters have a range of TSSs that cluster within a 20-bp interval, which we have named the transcription start region (TSR). When locating TSRs, overlap on the same strand was not allowed. In the control data sets, we found 91,354 host TSRs and 14,005 HCMV TSRs using the same cutoff of 20 reads per TSR. These results show that the newly replicated HCMV DNA is 2 orders of magnitude more permissive to the transcription machinery than host DNA. Because the number of TSRs found on the HCMV genome vastly exceeds the number of known genes and because the TSRs span more than 4 orders of magnitude in strength, it is unlikely that most produce biologically significant levels of mRNAs. Instead, as will be discussed below, it is more likely that the results found are due to the fact that the Pol II initiation machinery has easy access to newly replicated DNA late in infection.

The robustness and reproducibility of the PRO-Seq results were tested by comparing data sets generated by infection of HFF from two different donors with two related viral strains (the Towne varS and the Towne BAC recombinant virus). Libraries were prepared by two different individuals using subtly different library generation protocols almost a year apart. Data sets from infected cells (control and flavopiridol-treated cells) were normalized to the total number of reads, and correlation plots were generated from the read densities at each base position (212,000 bp) in the forward and reverse directions (Fig. 1B). The data covered almost 5 orders of magnitude, and Spearman coefficients were 0.83 to 0.85, demonstrating a high level of reproducibility even with the indicated biological and technical differences between the data sets.

**FIG 2:**
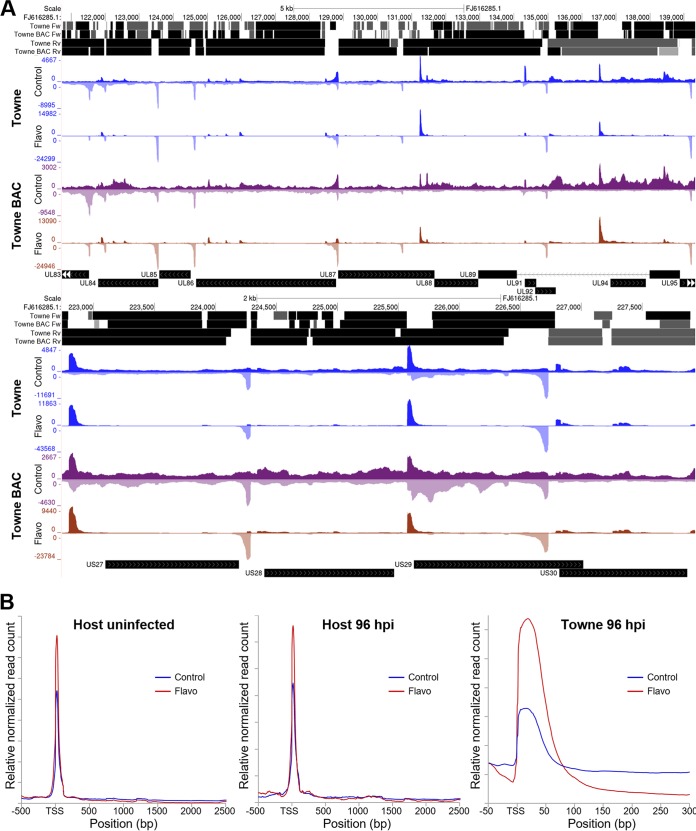
Prevalence of flavopiridol-sensitive productive elongation. (A) UCSC Genome Browser views of sample regions of PRO-Seq from two biologically distinct infections (Towne and Towne BAC) with ROPE analyses for forward (Fw) and reverse (Rv) reads derived from the difference between the control and flavopiridol (Flavo) data sets. Average reads across the ROPEs are broken into quartiles and shaded (black is the top 25%). (B) Metagene analyses from the host before (29,838 TSRs) and after (20,784 TSRs) infection with HCMV (1,211 TSRs) comparing control and flavopiridol data normalized across the region shown.

The two independent sets of data were analyzed using a method that we developed to identify regions of productive elongation (ROPEs). Matching control and flavopiridol data sets were read normalized before the flavopiridol signal was subtracted from the control signal. Regions of promoter-proximal pausing had negative values, and ROPEs were positive. We generated a track in which ROPEs are depicted as boxes for both the forward and reverse directions, with shading correlating with the relative amount of productive elongation. Figure 2A shows two representative regions of the HCMV genome with the PRO-Seq signals and ROPE analysis for the two sets of data. Visually, it is clear that the Towne BAC had more productive elongation compared to Towne. This is likely due to differences in cellular conditions during culturing. Fractional coverage of the genome was 70% forward and 89% reverse for Towne and 64% forward and 87% reverse for Towne BAC. Importantly, the regions identified for Towne and Towne BAC overlapped 77% in the forward and 95% in the reverse direction. A binomial test was used to determine that the overlap was statistically significant (*P* values were less than 2.2 × 10^−16^ for both forward and reverse data). Metagene analysis indicates a low, but significant, level of productive elongation in the uninfected host genome, as seen by an increase in short paused transcripts and a decrease in long productively elongating transcripts caused by flavopiridol treatment (Fig. 2B). Metagene analysis of HCMV demonstrated a higher level of productive elongation than seen for the average host gene. Evidently, Pol II pausing and elongation control by P-TEFb is broadly employed across the HCMV genome.

### Transcription of the MIE promoter/enhancer region.

**FIG 3:**
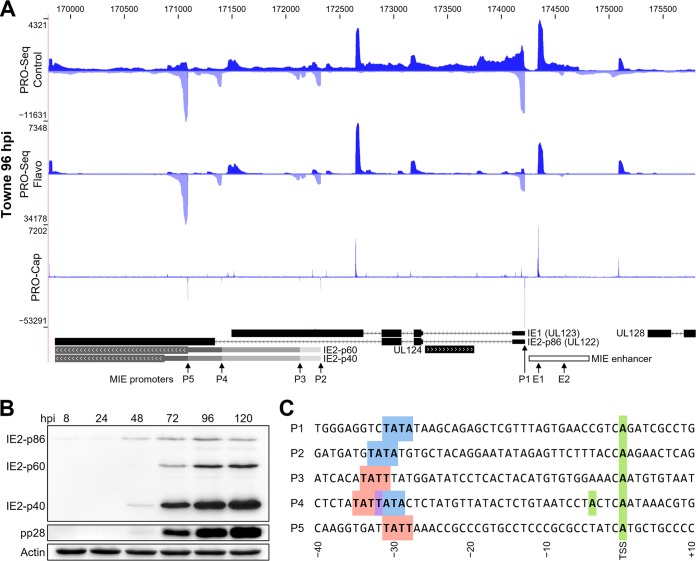
Regulated transcription of major immediate early (MIE) promoters. (A) PRO-Seq and PRO-Cap from HFF infected with HCMV strain Towne 96 hpi. All tracks range from 0 to the indicated number of reads and are not otherwise normalized. Coding regions (black, thick) from GenBank (accession number FJ616285.1), IE1- and IE2-p86 5′ untranslated regions (UTRs) (black, thin) and potential coding regions for IE2-p60 and IE2-p40 (gray) (33, 64), and the MIE enhancer region (white) (21) are identified. MIE promoter and enhancer TSSs are indicated. E1 is on the forward strand, while P1 to P5 and E2 are on the reverse strand. (B) Western blot of IE2 isoforms, late HCMV tegument protein pp28, and actin and 8% SDS-PAGE. (C) Base composition of MIE promoters P1 to P5. Highlighted are major TSSs (green) and TATA (blue) or TATT (red) elements starting 34 to 30 bp upstream. P4 has two major start sites: one presumably driven by TATA and the other driven by TATT.

An exploded view of the important MIE region provides an example of how PRO-Seq and PRO-Cap can be used to help annotate transcription start sites and reveal previously unknown transcription units (Fig. 3A). As expected, the precise locations of promoter-proximally-paused Pol II obtained by PRO-Seq from flavopiridol-treated infected cells closely mirrors the PRO-Cap results that locate positions of 5′ ends of capped RNAs. Either approach can therefore be used to pinpoint locations of transcription start sites, but PRO-Cap more accurately reflects the relative abundance of each TSS. As expected, the MIE promoter (labeled P1) produces transcripts in late infection that code for IE1 and IE2-p86. The two shorter IE2 isoforms, p40 and p60, are produced only in late infection (Fig. 3B) from downstream late promoters (31, 32). The P5 promoter abundantly produces transcripts for p40, consistent with previous reports (31–33). Transcripts for the p60 isoform likely arise from promoters P2 to P4. This might explain prior reports of multiple late IE2 mRNAs slightly larger than the p40 transcript (31) and failure of P4 mutations to completely eliminate p60 expression (33). The P3-to-P5 promoters each have a TATT element that is characteristic of viral late promoters (34). Like the MIE promoter, the P2 promoter has a TATA element upstream of the TSR. The P4 promoter is unusual in having partially overlapping TATT and TATA elements, which may be the basis for the two P4 TSSs separated by 4 bases. Potential promoters for UL124 on the opposite DNA strand are also evident.

The 500-bp enhancer immediately upstream of the MIE promoter (P1) is required for transcription of UL123 (IE1) and UL122 (IE2-p86) (35). This region is transcribed 96 hpi in both directions from two promoters, MIE E1 (forward) and E2 (reverse) (Fig. 3A). MIE E1-derived transcripts have not been previously described, but the amount of paused Pol II from E1 is close to half that produced by the MIE P1 promoter. E1-derived RNAs are not found in poly(A)^+^ RNA-Seq data sets for HCMV Merlin (26) or TB40E (25) strains at 72 hpi, suggesting that this RNA may be unstable. Both E1 and E2 have upstream TATT sequences, suggesting they may be driven by the late transcription machinery. E2 is the likely source of the HCMV ORF94 mRNA that was previously detected by PCR methods in an HCMV latency model (36) and in HFF at late times of productive infection (25, 37).

### Transcription of oriLyt.

**FIG 4:**
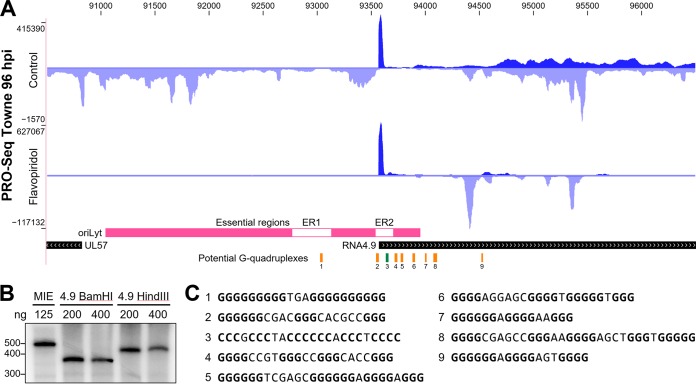
Regulated transcription around oriLyt and RNA4.9. (A) PRO-Seq from control and flavopiridol-treated HFF infected with HCMV strain Towne at 96 hpi. All tracks range from 0 to the indicated number of reads and are not otherwise normalized. Essential regions (white) within oriLyt (pink) (39) are indicated. Potential G quadruplexes are indicated (orange, forward strand; green, reverse strand). (B) HeLa nuclear extracts and the indicated amounts of soluble MIE (508-nt runoff) or the pUC19-RNA4.9 promoter template cut with BamHI (350-nt runoff) or HindIII (380-nt runoff) were preincubated for 30 min, pulsed for 30 s with limiting [α-^32^P]CTP, and chased for 5 min and subject to 6% urea-PAGE. (C) Forward-strand base composition of potential G quadruplexes.

One of the most surprising results of our initial study examining nascent HCMV transcripts was the discovery of a massive peak of promoter-proximally-paused Pol II in the RNA4.9 gene (Fig. 1 and 4). At 96 hpi of HFF with Towne, 36% of the total PRO-Seq reads were over the HCMV genome, and 8% of those were from transcripts paused downstream of the RNA4.9 promoter. Although very little is known about the function of this long noncoding RNA, it is expressed during lytic infection (26) and can be found at low levels in latent infections (38). Importantly, a set of overlapping 200-bp deletions was made across the region of HCMV encompassing oriLyt, and two essential regions (ERs) for viral replication were defined (39) (Fig. 4A, ER1 and ER2). Although, at the time, RNA4.9 had not been identified, our data now indicate that ER2 contains the TATA box and Inr of RNA4.9 as well as some transcribed downstream sequences (Fig. 4A). The RNA4.9 promoter was equally as active at driving transcription *in vitro* as the MIE promoter (Fig. 4B), in contrast to the much greater expression of RNA4.9 than that of the MIE promoter during viral infection. A lower level of transcription was detected from convergent promoters in the body of RNA4.9, heading in an antisense direction through the RNA4.9 promoter and into oriLyt.

Treatment of the infected cells with flavopiridol to inhibit P-TEFb had the expected effect of blocking productive elongation in the RNA4.9 gene and oriLyt region. Surprisingly, this increased the amount of Pol II engaged in promoter-proximal locations of the convergent promoters by 3 orders of magnitude. This increase is hard to explain simply by blocking the entry of Pol II into productive elongation in the convergent direction given that the rise in paused Pol II in the RNA4.9 gene was less than a factor of 2. We suggest that productive elongation from the RNA4.9 promoter interferes with initiation from the convergent promoters, thereby establishing a potential mechanism to regulate transcription through oriLyt that involves RNA4.9 transcription. This serves as an example of how PRO-Seq analysis of nascent transcripts can uncover transcriptional regulatory mechanisms that could not be detected by other methods. We also noticed that there was a high concentration of sequences with the potential to generate G quadruplexes around the RNA4.9 region. The presence of unusual structures is supported by evidence for persistent RNA/DNA hybrids in the oriLyt region (40). This might explain the unusually high level of Pol II paused just downstream of the RNA4.9 promoter. It is possible that transcription from this promoter and the resultant unusual nucleic acid structures play a role in regulating HCMV replication.

### Comparison of viral and host core promoters.

**FIG 5:**
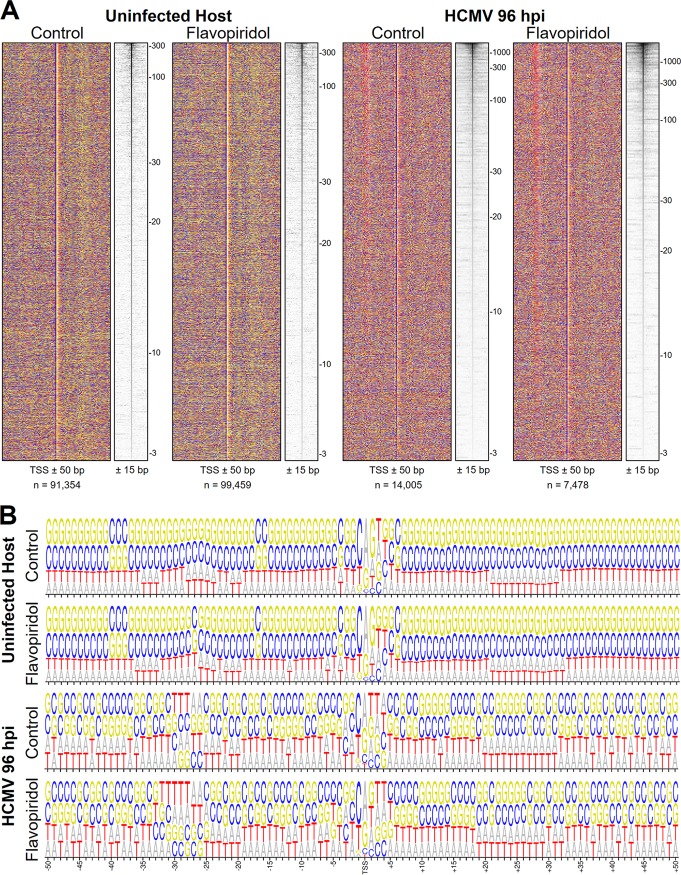
Sequence architecture of host and HCMV promoters. (A) Sequences and PRO-Cap signal around the MaxTSS within human control and flavopiridol TSRs, as well as of HCMV control and flavopiridol TSRs. Heatmaps were sorted by PRO-Cap signal of the MaxTSS. Bases: G, yellow; A, gray; T, red; C, blue. (B) Base composition probabilities for all TSRs in panel A from bp −50 to +50.

To characterize viral promoters and compare them to host promoters, we performed PRO-Cap on uninfected HFF and on Towne-infected HFF 96 hpi. Data from both control cells and flavopiridol-treated cells were analyzed. Sequence and TSS heatmaps were generated by aligning the TSS with the most reads in each TSR, the maximum TSS (MaxTSS), and displaying the sequence from −50 to +50 and the TSSs from −15 to +15 from each MaxTSS (Fig. 5A). Four colors, one for each base, were used in the sequence heatmaps. The TSRs were ranked in descending order by the number of reads in the MaxTSS as illustrated by the gradient signal from MaxTSSs in the TSS heatmaps. The TSS heatmaps were generated using a log_2_ transformation from the number of reads in each TSS. The scale beside the TSS heatmaps indicates the number of reads in the MaxTSSs. A number of features can be discerned from this visual analysis. First, each of the 4 sequence heatmaps have a vertical stripe in the center which covers the −1 and +1 base in each MaxTSS. This overlaps the Inr element that was recently defined as BBCA_+1_BW (41), where B stands for C, G, or T and W is A or T. Logos were created to display base composition probabilities of the −50 to +50 region for each set of TSRs (Fig. 5B). Although the Inr elements are similar (pyrimidine at −1 and purine at +1), HCMV displays less stringency for CA than the host does. Flavopiridol did not result in major sequence differences in the Inr region in the host, and the number of TSRs discovered in the host control and flavopiridol data were within 10% of each other (91,354 versus 99,459). However, almost twice as many TSRs were discovered in the HCMV control as in the flavopiridol data set. There was also a more relaxed Inr in the control logo than in flavopiridol logos. The discrepancy between the control and flavopiridol data sets will be addressed below. It is important to note that the earlier study (41) considered an order of magnitude fewer human TSSs (7,678) than we consider here, in part because that study examined only promoters in which nearly all TSSs were located within a few base pairs of the MaxTSS. The *inr* that we identify from host TSSs fits the consensus CA_+1_G sequence. The HCMV consensus sequence YR_+1_ was more relaxed. Positions +3 and +4 in the human and HCMV Inrs had a different base composition from that of the surrounding sequences, which had the general G/C richness, but the exact biases were different for human and HCMV.

Outside of the Inr, there were additional differences in the overall promoter architecture between host and HCMV. A region of T/A richness (red and white) is found upstream of many HCMV TSRs, especially in the flavopiridol data, but this element is difficult to discern in the host data (Fig. 5A). Similarly, a light band of purines (yellow and gray) can be seen in both host sequence heatmaps but not in those from HCMV. This downstream region has been implicated in interactions with subunits of the general initiation factor TFIID (42). It is also worth noting that from +20 to +30 in both host and CMV TSRs, A residues are more abundant than in the surrounding region. Interestingly, a purine-rich element in the +20-to-+30 region in *Drosophila melanogaster* has been linked to effective recruitment of NELF (43), which is consistent with promoter-proximal pausing at both host and viral genes.

It was concerning that the number of HCMV TSRs identified from control cells (14,005) was almost double the number from flavopiridol-treated cells (7,478). We generated tracks showing the TSRs found in the control and flavopiridol data sets. Although many TSRs completely or mostly overlapped between the two data sets, there were regions where many control TSRs did not have any corresponding TSRs in the flavopiridol data set. Several examples are shown in Fig. S1 in the supplemental material. TSRs with relatively high numbers of 5′ reads for the TSSs within them had highly similar patterns of TSSs, demonstrating high reproducibility between different data sets. However, in regions that did not contain TSRs in the flavopiridol data set, the read count was very low (Fig. S1; compare the PRO-Cap control and flavo signals in the lower sections of each panel). To determine if the discrepancy between the two data sets was due to low read counts in some regions, we performed a search for TSRs in both data sets using progressively higher read cutoffs from the 20-read default. This did not seem to be the problem, because at all values tested (up to the 100-read cutoff), the ratio of control to flavopiridol TSRs remained 2 to 1 (Table 1). We noticed that the regions containing control TSRs but no flavopiridol TSRs were also regions of high productive elongation. If the negative selection of uncapped transcripts used during the PRO-Cap library generation was not 100% efficient, 5′ ends from fragments of the transcripts from these regions might randomly survive the selection, enter into the PRO-Cap library, and then be falsely called TSRs. If that were true, then the transcripts from these regions would be longer on average than those from real TSRs, many of which are short because they are associated with paused Pol II. The average length from flavopiridol TSRs was 48 nucleotides (nt), and 93% of these had at least a 50% overlap with control TSRs. The average length of control TSRs with no corresponding flavopiridol TSR was about 120 nt. Therefore, we believe that regions of high productive elongation result in the calling of false TSRs and that the flavopiridol TSRs more accurately represent real TSRs. For all further analyses of HCMV data, the flavopiridol TSRs will be used. This artifact of the PRO-Cap method does not significantly affect the host analyses because the host genome has much lower levels of productive elongation and the depth of coverage of the host is lower than that of HCMV.

**TABLE 1:** Effect of TSR read cutoff on the ratio of control to flavopiridol TSRs^*a*^

No. of minimum reads/TSR	No. of TSRs in HFF		Control/Flavo
	Control	Flavo	
20	14,006	7,478	1.9
30	11,852	5,725	2.1
40	10,087	4,718	2.1
50	8,712	4,068	2.1
60	7,616	3,553	2.1
70	6,760	3,229	2.1
80	6,086	2,947	2.1
90	5,529	2,723	2.0
100	5,044	2,551	2.0

a Flavo, treated with flavopiridol. tsrFinder was used to discover the number of TSRs in both the control and flavopiridol data sets at the indicated number of minimum reads per TSR and the ratios of the numbers of control to flavopiridol TSRs were calculated.

10.1128/mBio.02047-18.1 FIG S1 Comparison of TSRs from the HCMV Towne control and flavopiridol data sets. TSR, PRO-Seq, and 5′ ends from PRO-Cap tracks are shown (forward direction only). Download FIG S1, PDF file, 0.3 MB. Copyright © 2019 Parida et al. 2019 Parida et al. This content is distributed under the terms of the Creative Commons Attribution 4.0 International license.

### Analysis of upstream elements.

**FIG 6:**
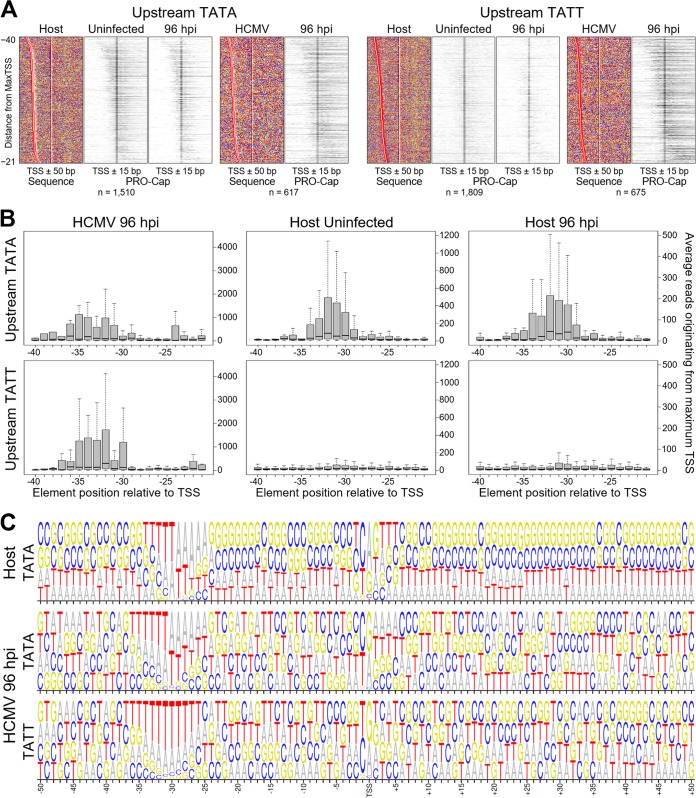
Influence of upstream promoter elements on Pol II initiation. (A) Human and 96-hpi HCMV TSRs with the initial base of TATA (left) or TATT (right) starting −40 to −21 bp upstream of the MaxTSS were selected, and sequences and PRO-Cap signal around the MaxTSS were plotted. Heatmaps were sorted by upstream promoter element distance. Bases: G, yellow; A, white; T, red; C, blue. (B) Influence of upstream TATA or TATT distance on Pol II initiation. The human and HCMV TSRs analyzed in panel A with the initial base of TATA (left) or TATT (right) starting −40 to −21 bp upstream of the MaxTSS were selected, and the average number of PRO-Cap reads originating from these MaxTSS were plotted using R boxplot. Box boundaries are drawn at the 25th and 75th percentiles, whiskers are 1.5 times the interquartile range, and the median is indicated. Outliers are not shown. (C) WebLogos for the indicated sets of genes.

The MIE promoter and many other HCMV promoters are driven by a TATA element containing an upstream element similar to that found in some host genes. However, many late viral genes are driven by promoters containing TATT, which is thought to be recognized by an alternative TATA-binding protein, UL87 (34). There is evidence that the HCMV late transcription factors UL87, UL49, UL88, UL91, UL92, UL95, and UL79 replace or augment the host general Pol II initiation and elongation factors in late transcription (34, 44, 45). We analyzed all HCMV and host TSRs with a TATA or TATT element with the first T of the elements located from −21 to −40 bp upstream of the MaxTSS (Fig. 6A). The percentages of host promoters containing TATA or TATT in this range were 1.6% and 2.0%, respectively (3.6% total), while the fractions for HCMV were about 5-fold higher (8.3% TATA and 9.0% TATT). Sequence heatmaps were generated and sorted by the distance of TATA or TATT from the MaxTSS in each TSR (Fig. 6A). Inr elements are still clearly visible in all groups. For both HCMV and the host, the utilization of all TSSs around the MaxTSS shows a shift in position that mirrors the position of TATA. TSSs upstream of the MaxTSS are favored when the TATA is far from the main TSS, and TSSs downstream of the MaxTSS are favored when TATA is close to the MaxTSS. This demonstrates that TATA is used as a positioning element. The ability of TATT to position start sites was less obvious in the host but clearly evident in HCMV. For host TATA-, HCMV TATA- and HCMV TATT-containing promoters, there was a strong correlation between the exact position of the upstream element and the strength of the TSS. The optimum location was between −30 and −34 for the host and −30 and −36 for HCMV (Fig. 6B). We have annotated all of the TATA and TATT motifs that start −30 to −36 upstream from the MaxTSS in each HCMV TSR, and these are viewable in the track hub. However, host promoters containing TATT did not display nearly as high a position-dependent increase in the strength of the MaxTSS as host promoters containing TATA. Importantly, during HCMV infection when the viral TATT promoters were very active, there was virtually no effect on TATT-containing promoters in the host. This demonstrates that the viral LTFs do not function significantly on host TATT elements. We also found that productive elongation occurred downstream of both TATA- and TATT-containing viral promoters (Fig. S2), indicating that the virus utilizes elongation control even for late gene transcription.

10.1128/mBio.02047-18.2 FIG S2 Metagene analysis of selected genes. Reads around TSSs from control and flavopiridol-treated HFF. Towne data sets with TATA (358 TSRs) (A) or TATT (411 TSRs) (B) in the region from bp –40 to –21 were normalized and plotted. Download FIG S2, PDF file, 0.2 MB. Copyright © 2019 Parida et al. 2019 Parida et al. This content is distributed under the terms of the Creative Commons Attribution 4.0 International license.

## DISCUSSION

Using PRO-Seq and PRO-Cap, we have analyzed transcription of the HCMV genome and compared it to transcription of the host genome, revealing a number of significant similarities and differences. As expected, the fraction of total mapped reads over the HCMV genome increased dramatically after viral DNA replication from 2% at 4 hpi with TB40E to 36% at 96 hpi with Towne. Considering the size and copy number of the viral genome, these values indicate that the density of viral transcription is much higher than for the host before and after viral replication has begun. At early and late times after infection, we found that inhibition of P-TEFb blocked productive elongation that otherwise covered the entire HCMV genome on both strands. It is now clear that Pol II elongation control is broadly operational across all viral transcription units, including transcription from promoters that may be driven by late viral transcription factors using TATT upstream elements. At 96 hpi, we found 7,478 HCMV TSRs, with a range of over 4 orders of magnitude of TSS strength, even though there are only several hundred HCMV genes. While some of these TSRs may simply result from the lack of transcriptional repression by chromatin, many might have functional significance. Other methods are needed to determine which of these TSRs produce significant levels of stable mRNAs. Another major difference between viral and host transcription is the lack of canonical divergent transcription on the virus genome. Host promoters in genes and enhancers are usually found as divergent pairs (14, 15, 46). HCMV promoters lack the standard spacing between the pairs seen in the host. Divergent transcription is seen by some as a mechanism to maintain promoter activity in a cellular environment that would otherwise assemble repressive chromatin over promoters (15). This may not be necessary during late infection, when viral genomes are rapidly produced and may lack extensive chromatin structure.

The PRO-Cap data allowed us to compare TSRs from the host and the virus at 96 hpi. While host Inrs were defined by a prominent CAG (–1 to +2), viral Inrs were a more relaxed YR (–1, +1), with a different preference at +2. Another striking difference between host and viral promoters was the prevalence of functional upstream elements (either TATA or TATT) in HCMV. The presence of TATT between −30 and −36 helped position the TSS and increase the strength of the promoter, but only on viral templates. This restriction of TATT function to viral templates is supported by previous results demonstrating colocalization of UL87 and other LTFs only to the region of infected cell nuclei containing replicated HCMV genomes (23). We also noted that the Inr for host TATA-containing genes matched the Inr for the average host gene, while the Inr for viral TATA was slightly different from the average viral Inr and from the viral TATT Inr.

Transcription of host genes takes place on chromatinized DNA, but how HCMV transcription is influenced by chromatin is less clear. The HCMV genome is delivered to the nucleus as essentially naked DNA early in infection, and late in infection, naked DNA is packaged into the viral capsid. The extent of chromatinization during transcription and replication of the viral genome has been more difficult to assess but is partly dependent on the type of cell. Based on a large number of studies and the results presented here, we propose a simple model that takes into account the general repressive nature of chromatin as well as the positive function of transcriptional activators on productive infection. Nondividing cells, such as contact-inhibited HFF, are highly conducive to productive infection partly because they do not enter S phase, during which canonical histones are being synthesized and nucleosomes assembled. Cycling HFF support only productive infection because early expression of IE2 and other viral proteins halt the cell cycle. Histone H1, which can rapidly exchange the host chromatin with the viral genome (47), acts as a repressor even during productive infection of contact-inhibited HFF. The viral IE2 protein negates the repressive effect of histone H1 (48, 49). The extensive deposition of histones to generate nucleosomes during host S phase blocks HCMV replication in all cell types, indicating that HCMV may need to have at least a fraction of its genomes devoid of nucleosomes. In nondividing cells, the replication of viral genomes and the pervasive transcription of the entire viral genome are potential means by which the virus contends with the threat of chromatin-mediated silencing. This explains why late genes are expressed only after viral genome replication has created new templates (50). Our data provide two new pieces of evidence for this model. The first is pervasive transcription, which generates one 20-bp TSR on average every 65 bp for the forward direction and every 61 bp for the reverse direction of the HCMV genome, a far higher density of transcription than the chromatinized host genome allows. The second is the inability of the viral late transcription factors to activate TATT-containing promoters in the host genome. While host promoter regions are relatively nucleosome free compared to the rest of the host genome, the chromatin environment at host promoters containing TATT is likely different than that found on the newly replicated viral genome. LTFs may not be able to compete with the host transcription factors and remodeling machinery for promoter occupancy. In support of this idea, UL87 is found only in regions of the nucleus containing HCMV DNA (23). HCMV may provide a model system to study the effects of chromatin on transcription that is impossible to achieve with uninfected mammalian cells.

## MATERIALS AND METHODS

### Cells and viruses.

Human foreskin fibroblasts (HFF) were isolated from deidentified discarded human foreskins. HFF were grown in T150 flasks at 37°C and 5% CO_2_ in 30 ml MEM (Gibco 11095-080) supplemented with 5% fetal bovine serum (FBS) and 1% penicillin-streptomycin (Gibco 15140-122). Stationary-phase cells were kept at 100% confluence with weekly medium changes. The medium was changed 1 day prior to infection. HCMV strains TB40E BAC4 (51, 52), Towne (varS), and Towne BAC (53) stocks were prepared, filter purified, and spun through sorbitol, and titers were determined as previously described (54). For titering by immunofluorescence, mouse anti-IE1/IE2-p86 (Millipore Sigma MAB810) and goat anti-mouse Alexa Fluor 555-conjugated IgG (Invitrogen A-21422) were used at 1:1,000. Infections were carried out at a multiplicity of infection (MOI) of 1 to 3. For PRO-Seq of TB40E-infected cells, T150 flasks of HFF were infected 4 h before harvest. All but 5 ml of medium was removed, and the remainder was inoculated with HCMV strain TB40E (MOI of about 3). One hour before harvest, 5 ml medium was collected, mixed with 5 µl DMSO or 1 mM flavopiridol (NIH AIDS Reagent Program 9925) dissolved in DMSO, and returned to cells (final concentrations, 0.1% DMSO ± 1 µM flavopiridol). For PRO-Seq and PRO-Cap of Towne-infected cells, T150 flasks of HFF were infected for 96 h before harvest. Immediately prior to infection, the growth medium was removed from the T150 flasks of HFF. The viral stock was diluted in the conditioned medium to the appropriate titer, and 6 ml of this inoculum was placed in each T150 flask of HFF. Viral adsorption was carried out for 90 min in a 5% CO_2_ incubator, the inoculum was removed, and unattached virus was washed away using conditioned medium. The Towne varS-infected cells were then grown in fresh medium, whereas the Towne BAC-infected cells were grown in conditioned medium. Mock-infected HFF were treated the same way as infected cells, but minus the virus. All but 6 ml of medium was removed from each flask 1 h prior to harvest, and 6 µl of DMSO or 1 mM flavopiridol in DMSO was added (final concentrations, 0.1% DMSO ± 1 µM flavopiridol).

### PRO-Seq and PRO-Cap.

All rapid nucleus isolation steps were performed on wet ice using ice-cold buffers as previously described (30). After infection and treatment of cells, T150 flasks were removed from the incubator, and over 20 s, medium was discarded; phosphate-buffered saline (PBS) was added, quickly mixed, and discarded, and 10 ml lysis buffer (20 mM HEPES, pH 7.6, 300 mM sucrose, 1% IGEPAL CA-630, 1 mM spermine, 1 mM spermidine, 1 mM EDTA, 1 mM dithiothreitol [DTT], 0.004 U/μl SUPERase-In [Ambion AM2696], 0.1% isopropanol-saturated phenylmethylsulfonyl fluoride [PMSF], and cOmplete EDTA-free protease inhibitor cocktail [Roche 11873580001]) was introduced. After a brief incubation on ice, lysis was confirmed by phase-contrast microscopy. Lysates were then scraped and transferred to 15-ml conical tubes. In 50-ml round-bottom polycarbonate Nalgene tubes (Thermo 3117-0500), lysates were then layered over 17-ml sucrose cushions (20 mM HEPES, pH 7.6, 1 M sucrose, 1 mM spermine, 1 mM spermidine, 0.1 mM EDTA, 1 mM DTT, 0.004 U/μl SUPERase-In, 0.1% isopropanol-saturated PMSF, and cOmplete EDTA-free protease inhibitor cocktail) and spun at 22,500 × *g* for 5 min. Pelleted nuclei were resuspended in 100 µl storage buffer (20 mM HEPES, pH 7.6, 5 mM magnesium acetate, 150 mM potassium acetate, 5 mM DTT, and 25% glycerol) and stored at −80°C.

For each experimental condition, 100-µl nucleus aliquots in storage buffer were heated to 37°C and then incubated for 10 min with 100 µl of a preheated reaction buffer containing 20 mM HEPES, pH 7.6, 5 mM magnesium acetate, 150 mM potassium acetate, 5 mM DTT, 1% Sarkosyl, 0.5 U/μl SUPERase-In, and 25 µM (each) biotin-11-ATP (PerkinElmer NEL544), -UTP (NEL543), -GTP (NEL545), and -CTP (NEL542). Reactions were stopped with 600 µl TRIzol LS (Ambion 10296028), and RNA was isolated, precipitated with 95% ethanol and 500 mM ammonium acetate, and washed with 70% ethanol. Pellets were resuspended in either 20 µl (TB40E) or 45 µl (Towne) RNase-free H_2_O, incubated at 65°C for 2 min, and then snap-cooled on ice. Towne samples were then split into two 20-µl aliquots: one for PRO-Seq and one for PRO-Cap. PRO-Seq samples were incubated on ice for 20 min with 5 µl 1 N NaOH and then quenched with 25 µl 1 M Tris, pH 7.8; PRO-Cap samples were instead incubated on ice with 30 µl RNase-free H_2_O. During the hydrolysis step, M-280 streptavidin Dynabeads (Invitrogen 11206D) were prepared (150 µl per library) by washing them twice with M-280 high-salt wash (50 mM Tris, pH 7.8, 2 M NaCl, 0.5% Triton X-100, and 1 mM EDTA) and then resuspending them in 150 µl per library of M-280 high-salt wash. Dynabeads were stored at 4°C with rotation until needed (no more than a few days).

After the hydrolysis step, all samples were incubated with 1 µl SUPERase-In and 50 µl washed Dynabeads at room temperature (RT) for 15 min with rotation. Beads were washed three times with 500 µl M-280 high-salt wash and twice with 500 µl M-280 low-salt wash (20 mM Tris, pH 7.8, 150 mM NaCl, 0.1% Triton X-100, and 1 mM EDTA). Beads were then resuspended with TRIzol LS, and RNA was isolated and precipitated. Pellets were resuspended in 8 µl 12.5 µM RNA adapter VRA3 (TB40E) or VRA3-4N (Towne), incubated at 65°C for 2 min, snap-cooled on ice, and incubated at 37°C for 4 h with 12 µl 1.67× Rnl1 mix (1.67 U/µl T4 RNA ligase 1 single-stranded RNA [ssRNA] [NEB M0204], 5/3× ligase reaction buffer, 1.67 mM ATP, 25% polyethylene glycol 8000 [PEG 8000], and 1.67 U/µl SUPERase-In). Ligated samples were then incubated with 30 µl RNase-free H_2_O and 50 µl washed Dynabeads at RT for 15 min with rotation. Beads were washed three times with 500 µl M-280 high-salt wash and twice with 500 µl M-280 low-salt wash. Beads were then resuspended in TRIzol LS, and RNA was isolated and precipitated. Pellets were resuspended in 10 µl RNase-free H_2_O, incubated at 65°C for 2 min, and snap-cooled on ice.

PRO-Cap samples were treated sequentially with RNA 5′ polyphosphatase, Terminator 5′-phosphate-dependent exonuclease, and shrimp alkaline phosphatase. First, samples were incubated at 37°C for 1 h with 10 µl 2× polyphosphatase mix (2 U/µl RNA 5′ polyphosphatase [Epicentre RP8092H] and 2× reaction buffer). RNA was isolated with TRIzol LS and precipitated, and pellets were resuspended in 10 µl RNase-free H_2_O, incubated at 65°C for 2 min, and snap-cooled on ice. Then, samples were incubated at 30°C for 1 h with 10 µl 2× Terminator mix (0.1 U/µl Terminator 5′-phosphate-dependent exonuclease [Epicentre TER51020], 2× reaction buffer A, and 2 U/µl SUPERase-In). RNA was isolated with TRIzol LS and precipitated, and pellets were resuspended in 10 µl RNase-free H_2_O, incubated at 65°C for 2 min, and snap-cooled on ice. Finally, samples were incubated at 37°C for 1 h with 10 µl 2× recombinant SAP (rSAP) mix (0.1 U/µl recombinant shrimp alkaline phosphatase [NEB M0371], 2× CutSmart buffer, and 2 U/µl SUPERase-In). RNA was isolated with TRIzol LS and precipitated, and pellets were resuspended in 10 µl RNase-free H_2_O, incubated at 65°C for 2 min, and snap-cooled on ice. These steps were skipped for PRO-Seq samples.

All samples were incubated at 37°C for 1 h with 10 µl 2× RppH mix (0.5 U/µl RNA 5′ pyrophosphohydrolase [NEB M0356], 2× ThermoPol buffer, and 1 U/µl SUPERase-In). PRO-Seq samples were then incubated at 37°C for 1 h with 80 µl 5/4× T4 polynucleotide kinase (PNK) mix (0.313 U/µl T4 PNK [NEB M0201], 5/4× T4 PNK buffer, 1.25 mM ATP, and 0.625 U/µl SUPERase-In). PNK treatment was skipped for PRO-Cap samples. RNA was isolated with TRIzol LS and precipitated, and pellets were resuspended in 8 µl 12.5 µM RNA adapter VRA5 (TB40E) or VRA5-4N (Towne), incubated at 65°C for 2 min, snap-cooled on ice, and incubated at 37°C for 4 h with 12 µl 5/3× Rnl1 mix. Ligated samples were then incubated with 30 µl RNase-free H_2_O and 50 µl washed Dynabeads at RT for 15 min with rotation. Beads were washed three times with 500 µl M-280 high-salt wash and twice with 500 µl M-280 low-salt wash. Beads were then resuspended with TRIzol LS, and RNA was isolated and precipitated. Pellets were resuspended in 10 µl 2× reverse transcription primer mix (5 µM RP1 and 1 mM deoxynucleoside triphosphate [dNTP] mix [NEB N0447]), incubated at 65°C for 2 min, snap-cooled on ice, and incubated in a thermal cycler with 10 µl 2× SuperScript IV (SSIV) reverse transcriptase mix (20 U/µl SSIV [Invitrogen 18090050], 2× SSIV buffer, 10 mM DTT, and 2 U/µl SUPERase-In) at 45°C for 15 min, 50°C for 40 min, 55°C for 10 min, and 70°C for 15 min. Reverse-transcribed samples were in volumes up to 26 µl with RNase-free H_2_O, and 2 µl was used to test PCR conditions as described earlier (29). Final PCR mixtures were 50 µl and contained the remaining sample, 0.04 U/µl Phusion high-fidelity (HF) DNA polymerase (NEB M0530), 1× HF buffer, 1 M betaine (Sigma B0300), 250 µM dNTP mix, 0.25 µM RP1, and 0.25 µM barcoded index primer (RPI-1 through RPI-8). Libraries were purified using a MinElute PCR purification kit (Qiagen 28004) and size selected for 135 to 600 bp using a BluePippin 2% agarose gel cassette (Sage Science BDF2010). Sequencing was performed by the Iowa Institute of Human Genomics on an Illumina HiSeq 4000 using 150-bp paired-end reads.

For the PRO-Seq replicate data sets from Towne BAC-infected cells, preparation of sequencing libraries was carried out with the following variations to the aforementioned protocol. Before the nuclear run-on with biotinylated NTPs, nuclei were briefly pelleted and storage buffer was removed. Nuclei were resuspended into 40 µl a buffer containing 20 mM HEPES, 5 mM MgCl_2_, 100 mM KCl, 5 mM DTT, and 0.6 U/μl SUPERase-In). Nuclear run-on reactions were carried out in the presence of a 20 µM concentration (each) of the biotinylated NTPs in a final volume of 60 µl. Following the hydrolysis step for PRO-Seq, reactions were quenched with Tris, pH 6.8. All PCR steps were carried out using KAPA HiFi HotStar ReadyMix (KAPA Biosystems KK2601) per the manufacturer’s instructions.

### Oligonucleotides for PRO-Seq and PRO-Cap.

The oligonucleotides for PRO-Seq and PRO-Cap were as follows: VRA3, /5Phos/rGrArUrCrGrUrCrGrGrArCrUrGrUrArGrArArCrUrCrUrGrArArC/3InvdT/; VRA3-4N, /5Phos/rNrNrNrNrGrArUrCrGrUrCrGrGrArCrUrGrUrArGrArArCrUrCrUrGrArArC/3InvdT/; VRA5, rCrCrUrUrGrGrCrArCrCrCrGrArGrArArUrUrCrCrA; VRA5-4N, rCrCrUrUrGrGrCrArCrCrCrGrArGrArArUrUrCrCrArNrNrNrN; RP1, AATGATACGGCGACCACCGAGATCTACACGTTCAGAGTTCTACAGTCCGA; RPI-1, CAAGCAGAAGACGGCATACGAGATCGTGATGTGACTGGAGTTCCTTGGCACCCGAGAATTCCA; RPI-2, AAGCAGAAGACGGCATACGAGATACATCGGTGACTGGAGTTCCTTGGCACCCGAGAATTCCA; RPI-3, AAGCAGAAGACGGCATACGAGATGCCTAAGTGACTGGAGTTCCTTGGCACCCGAGAATTCCA; RPI-4, AAGCAGAAGACGGCATACGAGATTGGTCAGTGACTGGAGTTCCTTGGCACCCGAGAATTCCA; RPI-5, AAGCAGAAGACGGCATACGAGATCACTGTGTGACTGGAGTTCCTTGGCACCCGAGAATTCCA; RPI-6, AAGCAGAAGACGGCATACGAGATATTGGCGTGACTGGAGTTCCTTGGCACCCGAGAATTCCA; RPI-7, AAGCAGAAGACGGCATACGAGATGATCTGGTGACTGGAGTTCCTTGGCACCCGAGAATTCCA; and RPI-8, AAGCAGAAGACGGCATACGAGATTCAAGTGTGACTGGAGTTCCTTGGCACCCGAGAATTCCA.

### Data analysis.

Sequences were first trimmed using trim_galore 0.4.4 (https://github.com/FelixKrueger/TrimGalore) and then aligned with the UCSC hg38 assembly using bowtie 1.2 (55). Unmapped reads were saved and separately aligned to the HCMV strain Towne genome (GenBank accession number FJ616285.1) (56, 57). Towne-infected samples were deduplicated using dedup, a new program that collapses identical mapped reads with redundant unique molecular identifiers (https://github.com/P-TEFb/dedup). Pileup (PRO-Seq) or 5′ site (PRO-Cap) tracks were generated using bedtools 2.26 (58) and displayed on the UCSC Genome Browser (59). HCMV tracks were displayed using a custom track hub (60). Transcription start regions were identified with tsrFinder, a new program that evaluates 5′ PRO-Cap read densities within user-defined intervals (https://github.com/P-TEFb/tsrFinder). Heatmaps were generated with python 2.7 scripts and the graphics package in R. Sequence logos were generated with weblogo 3.6.0 (61). The sequence and TSS heatmaps were sorted based on their MaxTSS read counts in descending order. Sequence heatmaps and logos were colored as follows: A was gray (hex code #bbbbbb), T was red (hex code #ff0000), G was yellow (hex code #dddd00), and C was blue (hex code #2222ff). The read counts for each TSS in the TSS heatmaps were log_2_ transformed. A gradient hex color code list of size 256, ranging between white and black, was generated using the colorRampPalette function in R. This list was used by the image function in the R graphics package to assign each TSS log_2_-transformed read count in the TSS heatmaps with an appropriate color code based on their rank and subsequently plot them. A scale showing the observed read count for MaxTSS at specific intervals was manually added adjacent to the TSS heatmaps for convenient visualization of the data distribution. Base composition probabilities were computed, per base pair, from the sequences used in the sequence heatmap and plotted as sequence logos.

Sequence and TSS heatmaps for host and HCMV sequences containing a TATA and TATT subsequence between −40 and −21 bp from their MaxTSS were drawn by following the techniques mentioned above, except that nucleotide A in the sequence heatmaps was white (hex code #FFFFFF) instead of gray for improved contrast. These MaxTSSs were further used to draw TSS heatmaps using the 96-hpi host PRO-Cap data to allow comparison between uninfected and infected host data sets. Next, MaxTSSs were grouped based on their starting base pair position for the TATA and TATT subsequences within the −40- and −21-bp window. The Boxplot function in the R graphics package was used to draw boxplots with their respective medians (black), describing the read count distribution for the MaxTSSs within each group. These plots provided information on the optimum position or range of positions for these subsequences based on the rank of their medians. This information was used to narrow down sequences from the above list to generate two new lists, such as uninfected host sequences containing the TATA subsequence within the −34- and −30-bp window and HMCV sequences containing the TATA and TATT subsequences within the −36- and −30-bp window from their MaxTSSs. Finally, the sequence logos were drawn for all three lists using the same technique and tools as described earlier to allow comparison between them. PRO-Seq and PRO-Cap data sets can be obtained from GEO (accession number GSE113394 [use token wtsteucevpwbfsn to access]). The HCMV track hub is available on GitHub (https://github.com/P-TEFb/trackHub_HCMV).

### Correlation analysis.

Quality-controlled Towne and Towne BAC PRO-Seq reads for DMSO and flavopiridol samples were mapped to their respective genomes. The genomeCoverageBed program under bedtools was used to generate read coverage for each genomic position per sample and strand independently. Genomic positions with at least 1 read in both genomes were retained for further analysis. Each position was normalized by dividing the specific read coverage with a scaling factor. The factor was computed as the sum of read coverage of all genomic positions/1,000,000. This scaling was performed to normalize the area under the curve for Towne and Towne BAC data sets. Finally, a Spearman correlation coefficient was computed between Towne and Towne BAC samples using the R software package, and scatterplots were made using Microsoft Excel.

### ROPE analysis.

A region of productive elongation (ROPE) was defined as a contiguous stretch of positive genomic positions generated after subtracting scaled control data from flavopiridol data. A ROPE size of at least 21 bp was maintained to avoid erroneous regions from further analysis. ROPE calling was performed per strand of Towne- and Towne BAC-associated samples, independently. A Jaccard index was calculated to compare the percentages of overlap of ROPE regions between Towne and Towne BAC data sets. Additionally, this analysis was performed to generate the percent coverage between ROPE regions and their respective genomes. Fifty base pairs from the beginning of RNA 4.9 was excluded from the forward strands of all our samples due to inaccurate measurement of read depth in this region. Additional regions of missing DNA were excluded from further analysis as follows: (i) US2 to US11 (DNA was missing in Towne BAC), where the start was 197143 and the end was 206033 (removed from the percent overlap analysis); (ii) UL144 to UL150 (both genomes had missing DNA), where the start was 180887 and the end was 193884 (removed from the percent coverage analysis); and (iii) UL36 (DNA was missing in Towne), where the start was 48451 and the end was 48995 (removed from the percent overlap analysis). To determine if the percent overlap did not occur by random chance, we computed a *P* value using the binom.test function in the R software package. In the percent overlap computation, our data contained categories as follows: category a contained PRO-Seq reads covering a genomic position in both Towne and Towne BAC samples, and category b contained PRO-Seq reads covering a genomic position unique to Towne samples or Towne BAC samples but not both. We called category a a success and assigned it a probability of 0.3 under uniform distribution where all categories have an equal chance of occurring. Additionally, a two.sided alternative hypothesis and 95% confidence interval were chosen to perform this test.

A bed file consisting of 9 columns with the chromosome name, ROPE start, ROPE end, region number, ROPE region score, strand, ROPE start, ROPE end, and gray color, in this order, was made for control and flavopiridol samples under Towne and Towne BAC sample groups. These ROPE track bed files were uploaded and displayed under our UCSC hub FJ616285.1 assembly. A score was assigned to each ROPE region. This score was computed as the logarithm of the average of the differences between the scaled DMSO read coverage and the scaled flavopiridol read coverage. Next, ROPE regions were sorted in ascending order, categorized into quartiles, and colored based on their score, as follows: the first quartile was light gray (hex code #C0C0C0), the second quartile was dark gray (hex code #808080), the third quartile was very dark gray (hex code #404040), and the fourth quartile was black (hex code #000000).

### Metagene analyses.

Host metagene profiles were generated using a customized python 2.7 script by centering the MaxTSS in each of the 91,354 uninfected and 99,459 infected host control TSRs. TSRs associated with chromosomes 1 to 22, X, and Y discovered from our PRO-Cap host control data sets were used for this analysis. A region of a window (−500 to +3,000 bp) from the MaxTSSs on the plus strand and a window (−3,000 to +500 bp) from the MaxTSSs on the minus strand were used to generate read counts per base pair for each window from their respective control and flavopiridol PRO-Seq data sets. The minus strand windows were flipped to maintain the same order as the windows on the plus strand for uniformity while they were plotted. Windows that contained another MaxTSS with an equal or greater PRO-Cap read count than their center MaxTSS’s PRO-Cap read count or containing a letter N in their sequence were excluded from further analysis due to misrepresentation of productive elongation pattern. Additionally, we removed those TSRs that overlapped our list of blacklisted regions, which included microRNA (miRNA), ribosomal RNA (rRNA), small cytoplasmic RNA (scRNA), small nuclear RNA (snRNA), small nucleolar RNA (snoRNA), small Cajal body-specific RNA (scaRNA), and transfer RNA with 50 bp padded to their start and end sites (hg38.GencodeV27.miRNA-rRNA-scRNA-snRNA-snoRNA-rRNA-scaRNA-tRNA.bed is a file made with the “transcript_biotype” tags mentioned above from the ncRNA file, available under “FTP Download, Species: Human, ensemble,” and hg38.tRNAscan.bed is available in the UCSC Table Browser under the group “Genes and Gene Predictions” and the track “tRNA Genes”). Totals of 29,838 uninfected host and 20,784 infected control TSRs survived our filtering criteria and were used for further analysis. A sum total of read counts per base pair of all 3,500-bp windows were computed independently. These exact TSRs and their 3,500-bp windows were used to generate per base pair a sum total of read counts from the uninfected host and infected flavopiridol data sets. The total read coverage per base pair in the 3,500-bp window per sample per sample group was normalized by dividing it with the sum total of the respective column to keep the area under the curve equal to 1 for all samples. Finally, the normalized data were plotted using Microsoft Excel. These plots were used to compare productive elongation patterns between different host samples.

HCMV metagene profiles were generated using a customized python 2.7 script by centering the MaxTSS in each of the 7,478 HCMV flavopiridol TSRs discovered from our PRO-Cap Towne flavopiridol data. A region of a window (−50 to +300 bp) from the MaxTSSs on the plus strand and a window (−300 to +50 bp) from the MaxTSSs on the minus strand was searched to generate read counts per base pair for each window from the PRO-Seq control and flavopiridol data. Minus-strand windows were flipped, and the same filtering criteria from the host metagene analysis, with the exception of the blacklisted region filtering step, was applied to avoid misrepresentation of productive elongation pattern. Additionally, we ignored PRO-Seq data falling under RNA 4.9 (the start was 93569, the end was 98452), RNA 1.2 (the start was 2217, the end was 4694), and RNA 2.7 (the start was 6367, the end was 7392) for this analysis, due to the inaccuracy of read counts in these regions. A total of 1,211 flavopiridol TSRs survived our filtering criteria and were used for further analysis. A sum total of read counts per base pair of all 350-bp windows in control and flavopiridol samples was computed independently. Total read counts per base pair in this window per sample were normalized by dividing them by the sum total of their respective column and plotted using Microsoft Excel. This plot was used to compare productive elongation patterns between host and CMV samples. Similarly, metagene analyses were performed only using HCMV flavopiridol TSRs that contained a TATA sequence (358 TSRs) and a TATT sequence (411 TSRs) within a region from bp −40 to −21 from their MaxTSSs. This plot was used to compare productive elongation patterns between CMV samples containing TATA and TATT in their upstream regions.

### Western blotting.

For Western blotting of Towne-infected cells, all cells were infected at the same time (MOI of 1) and whole-cell extracts were collected at 8, 24, 48, 72, 96, or 120 h after infection. Western blotting of whole-cell extracts was performed using methods reported previously (54). Proteins were fractionated by sodium dodecyl sulfate-polyacrylamide gel electrophoresis (SDS-PAGE) on 8% Tris-glycine gels and transferred to Amersham Protran 0.45-µm nitrocellulose membranes (GE Healthcare Life Sciences 10600002). Blots were incubated overnight at 4°C with IE2 primary antibody MAB8140 in PBS with Tween 20 (PBST) containing 5% dried milk, incubated with the goat anti-mouse peroxidase-conjugated immunoglobulin G (IgG) F(ab′)_2_ fragment (Jackson ImmunoResearch 115-036-006) at room temperature for 1 h, and treated with SuperSignal West Femto maximum-sensitivity substrate (Thermo Scientific SJ257615) before being imaged on a UVP ChemStudio (Analytik Jena).

### *In vitro* transcription.

Transcription reactions were performed as previously described (3, 62). To generate the RNA 4.9 promoter template, HCMV plasmid B (63) was digested with EcoRI and BamHI, and the resulting 1.2-kb fragment was gel purified and ligated into an empty pUC19 cloning vector. This pUC19-RNA4.9 plasmid was grown in Escherichia coli and validated by sequencing. Preinitiation complexes were formed at room temperature for 30 min in 12-µl reaction mixtures containing the amounts of the soluble MIE promoter template (508 nt runoff) or pUC19-RNA4.9 promoter template cut with BamHI (350 nt runoff) or HindIII (380 nt runoff) indicated in the figures, 1 µl of HeLa nuclear extract, 20 mM HEPES, pH 7.6, 60 mM potassium acetate, 5 mM magnesium acetate, 1 mM DTT, 0.1% DMSO, and 0.5 U/μl SUPERase-In. Initiation was achieved by introducing 0.146 μM [α-^32^P]CTP (PerkinElmer BLU008H001MC) and 500 μM cold ATP, UTP, and GTP (2-µl addition). After 30 s, elongation complexes were chased for 5 min by introducing 1.25 mM cold CTP (2-µl addition). Reactions were stopped with 20 mM EDTA, and radiolabeled transcripts were phenol extracted, precipitated with 95% ethanol and 500 mM ammonium acetate, and separated by 6% urea-PAGE.

### Data availability.

A track hub for the HCMV Towne assembly has been made available on GitHub (https://github.com/P-TEFb/trackHub_HCMV). PRO-Seq and PRO-Cap raw data sets and bigwig files can be accessed under GEO (accession number GSE113394). TsrFinder, a new program that evaluates 5=PRO-Cap read densities within user-defined intervals and calls transcription start regions, can be accessed under GitHub (https://github.com/P-TEFb/tsrFinder). Dedup, a new program that collapses identical mapped reads with redundant unique molecular identifiers, can be accessed under GitHub (https://github.com/P-TEFb/dedup).
