# Supplementary material for: Nucleotide Resolution Comparison of Transcription of Human Cytomegalovirus and Host Genomes Reveals Universal Use of RNA Polymerase II Elongation Control Driven by Dissimilar Core Promoter Elements
Source: mBio. 2019 Feb 12;10(1):e02047-18. doi: 10.1128/mBio.02047-18 (PMC6372792; doi:10.1128/mBio.02047-18)
Supplement: FIG S1 [file mBio.02047-18-sf001.pdf]

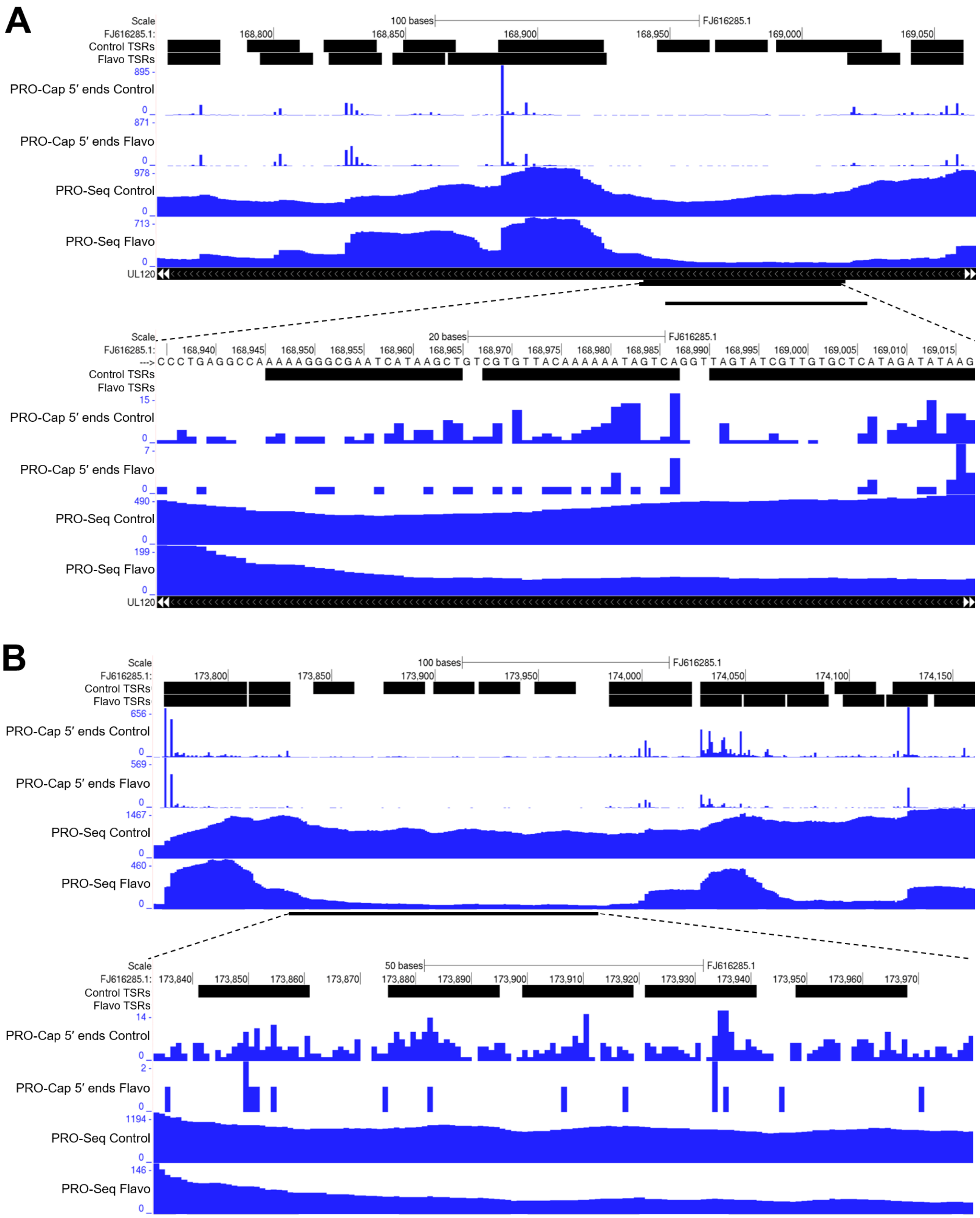

Fig. S1. Comparison of TSRs from the HCMV Towne control and flavopiridol datasets. TSR, PRO-Seq and 5′ ends from PRO-Cap tracks are shown (forward direction only).
