# Supplementary material for: Nucleotide Resolution Comparison of Transcription of Human Cytomegalovirus and Host Genomes Reveals Universal Use of RNA Polymerase II Elongation Control Driven by Dissimilar Core Promoter Elements
Source: mBio. 2019 Feb 12;10(1):e02047-18. doi: 10.1128/mBio.02047-18 (PMC6372792; doi:10.1128/mBio.02047-18)
Supplement: FIG S2 [file mBio.02047-18-sf002.pdf]

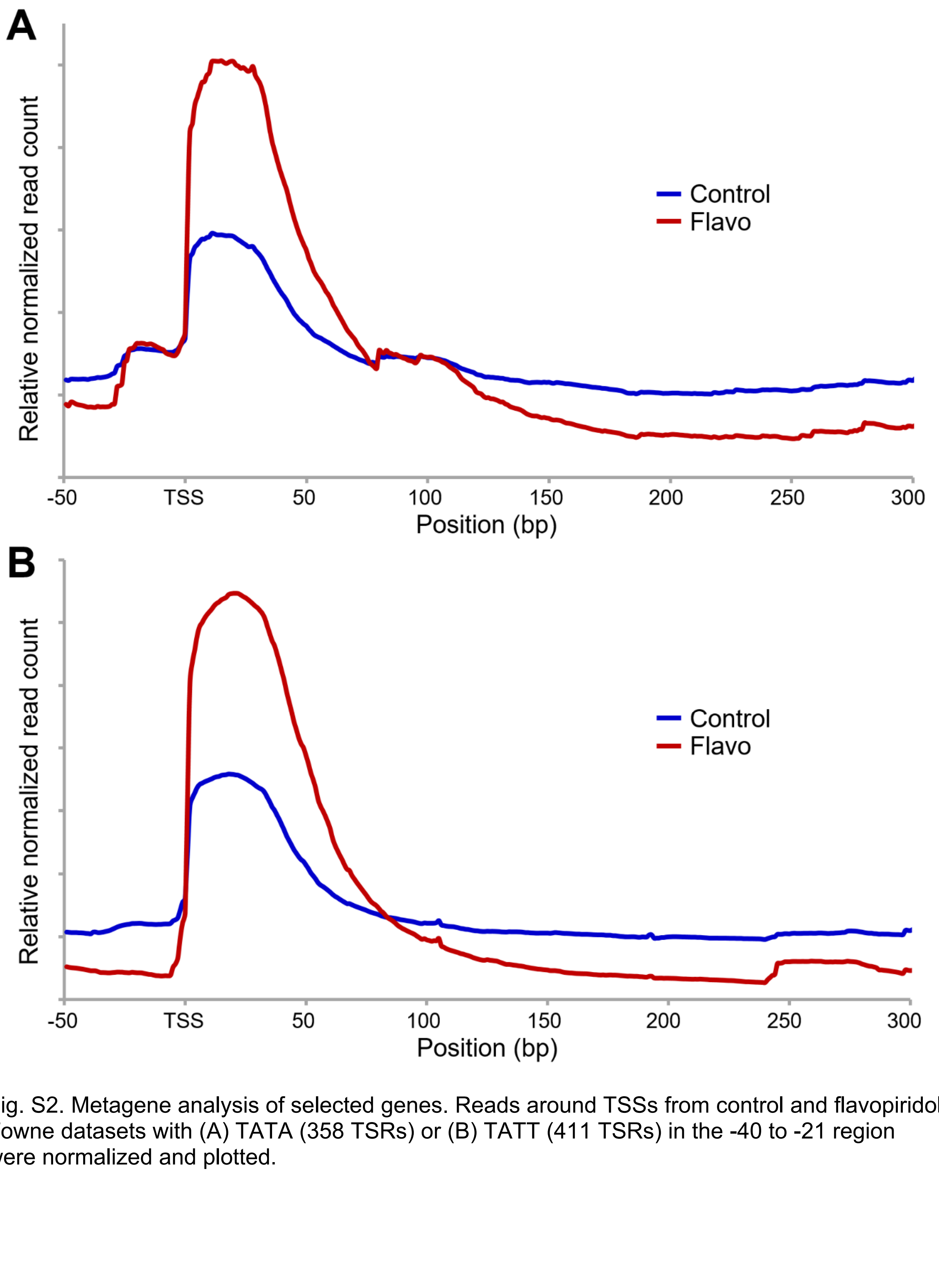

Fig. S2. Metagene analysis of selected genes. Reads around TSSs from control and flavopiridol Towne datasets with (A) TATA (358 TSRs) or (B) TATT (411 TSRs) in the -40 to -21 region were normalized and plotted.
